# Differences in Immune Checkpoints Expression (TIM-3 and PD-1) on T Cells in Women with Recurrent Miscarriages—Preliminary Studies

**DOI:** 10.3390/jcm10184182

**Published:** 2021-09-16

**Authors:** Michał Zych, Aleksander Roszczyk, Monika Kniotek, Filip Dąbrowski, Radosław Zagożdżon

**Affiliations:** 1 Department of Clinical Immunology, Transplantation Institute, Medical University of Warsaw, Nowogrodzka 59, 02-006 Warsaw, Mazovian Voivodeship, Poland; michal.zych@wum.edu.pl (M.Z.); monika.kniotek@wum.edu.pl (M.K.); radoslaw.zagozdzon@wum.edu.pl (R.Z.); 2 1st Department of Obstetrics and Gynecology, Medical University of Warsaw, Starynkiewicza 1, 02-015 Warsaw, Mazovian Voivodeship, Poland; filip.dabrowski@wum.edu.pl; 3 Department of Gynecology and Obstetrics, Medical University of Silesia, Medykow 14, 40-752 Katowice, Silesian Voivodeship, Poland; 4 Club35, Polish Society of Obstetricians and Gynecologists PTGiP, Cybernetyki7F/87, 02-677 Warsaw, Mazovian Voivodeship, Poland; 5 Department of Immunology, Transplantology, and Internal Diseases, Medical University of Warsaw, Nowogrodzka 59, 02-006 Warsaw, Mazovian Voivodeship, Poland

**Keywords:** immune checkpoints, miscarriage, pregnancy loss, PD-1, RSA, recurrent spontaneous abortion, TIM-3

## Abstract

Background: Immune checkpoints are molecules that regulate the function of immune cells and control inflammation processes. An important role in this regard is played by TIM-3/Gal-9 and PD-1/PDL-1 interactions. Previous research performed in a mouse model of pregnancy loss confirmed that blocking TIM-3 could induce fetal loss. Similarly, the PD-1 molecule maintains protective interactions between the mother’s immune cells and the fetus. The purpose of this study was to assess the expression of these molecules on a range of T lymphocyte subpopulations from non-pregnant women with recurrent spontaneous abortion (RSA) versus healthy fertile women. Methods: PBMCs were isolated by gradient centrifugation of blood obtained from 12 healthy women and 24 women with RSA and immediately stained for flow cytometry analysis. Standard immunophenotyping of PBMC was performed with the antibodies against classical lymphocyte markers: CD3, CD4, CD8, and CD56. Immune checkpoints were investigated using antibodies against PD-1(CD279) and TIM-3(CD366). Results: We found that expression of TIM-3 was significantly decreased on CD8+ T lymphocytes in the RSA group, and expression of PD-1 was upregulated on CD4+ T lymphocytes in the RSA group in comparison to the healthy controls. Conclusions: Considering our findings, therapeutic intervention towards immune checkpoints may be a promising treatment option for recurrent spontaneous abortion.

## 1. Introduction

Immune checkpoints (ICPs), negative (co-inhibitory) or positive (co-stimulatory), play an important role in the regulation and maintenance of immune homeostasis. They are extensively studied in the context of the re-establishment of immune reactivity to cancer cells; however, they also play a substantial role in numerous biological processes [1]. Co-inhibitory receptors binding with ligands may actively deliver inhibitory signals to mute T cells’ activation or counterbalance effects of positive signals delivered to them [2,3]. Moreover, co-inhibitory receptors promote the state of anergy in T lymphocytes that eventually leads to immune tolerance [3]. Negative ICPs prevent exaggerated effector T cell activation, which may eventually result in autoimmunity [2]. Co-inhibitory molecules can also indirectly affect T lymphocytes by promotion of the suppressive attributes of regulatory T cells and by regulating the ability of antigen-presenting cells (APCs) to prime T lymphocytes [2,4]. Distinct ICPs and their path of activation by ligands may control the secretion of various cytokine productions from T cells [2,5].

Immune checkpoints, such as T cell immunoglobulin-3 (TIM-3) and programmed cell death protein 1 (PD-1), are co-inhibitory receptors expressed on the surface of T lymphocytes (cytotoxic, helper, and regulatory), monocytes, macrophages, dendritic cells, and abundantly on natural killer (NK) cells [6,7]. TIM-3 is a type I transmembrane protein that is primarily described as a specific marker for T helper cells type 1 (Th1) and T cytotoxic cells type 1 (Tc1). There are two main ligands for TIM-3: C-type lectin galectin-9 (Gal-9) and CEACAM1 protein [8,9]. Galectin-9 binds to the N-linked sugar moieties in the TIM-3 IgV domain. The interaction leads to the activation of the apoptotic pathway in Th1 and Tc1 cells [9,10]. CEACAM1 is co-expressed together with TIM-3 on the surface of T lymphocytes. Cis-interaction between these molecules triggers the inhibitory function of TIM-3 [2]. Trans-interaction TIM-3–CEACAM1 also suppresses T cell function [8]. Two more ligands for TIM-3, phosphatidyl serine (PtdSer) and high mobility group protein B1 (HMGB1), were also described [11,12,13]. Numerous studies have shown that the TIM-3/Gal-9 pathway is involved in the regulation of immune responses and induction of tolerance during pregnancy [14,15,16]. Gal-9 is vastly expressed on decidual and trophoblast cells. The engagement of TIM-3 with Gal-9 is involved in the regulation of maternal tolerance toward trophoblast cells by inducing the exhaustion or apoptosis of effector T cells [17,18]. Studies performed on mice showed that blockade of the Tim-3/Gal-9 interaction resulted in an increment of Th1 cytokines released at the maternal–fetal interface and fetal rejection [19]. Alteration in Tim-3 expression has been reported in studies concerning women with pregnancy loss and preeclampsia [19,20,21,22,23]. 

**Figure 1:**
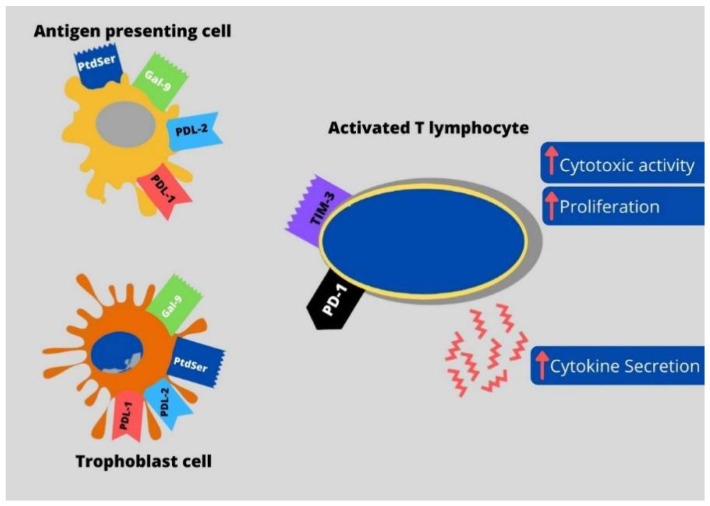
Distribution of immune checkpoints and their ligands on antigen-presenting cells (APCs), trophoblast cells, and activated T lymphocytes: PD-1—programmed cell death protein 1, TIM-3—T cell immunoglobulin-3, PDL1—programmed death-ligand 1, PDL-2—programmed death-ligand 2, PtdSer—phosphatidyl serine, Gal-9—galectin-9.

**Figure 2:**
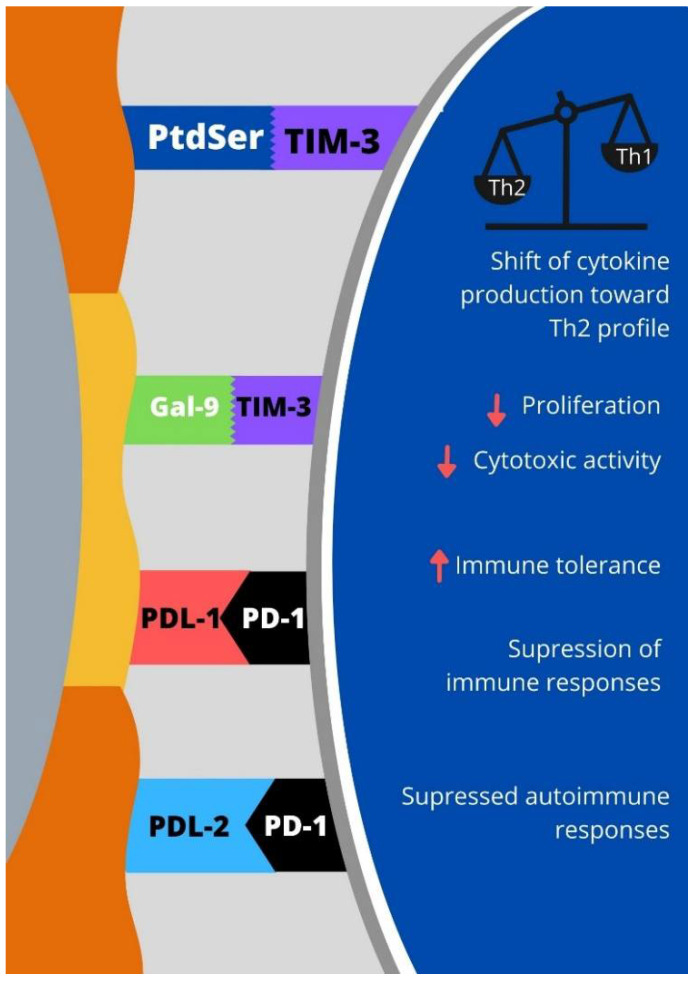
Interactions and their effects between immune checkpoints and their ligands expressed on immunocompetent cells with effector mechanism: PD-1—programmed cell death protein 1, TIM-3—T cell immunoglobulin-3, PDL-1—programmed death-ligand 1, PDL-2—programmed death-ligand 2, PtdSer—phosphatidyl serine, Gal-9—galectin-9.

Programmed cell death protein 1 (PD-1) is a transmembrane protein that belongs to the B7-CD28 family [24,25]. PD-1 is expressed on the membrane of activated immune cells such as helper T cells, cytotoxic T cells, natural killer T-cells (NKT) cells, and regulatory T cells. Moreover, PD-1 molecules are expressed on B lymphocytes, monocytes, and dendritic cells [24,26,27]. Interactions of PD-1 with PDL-1 and PDL-2 induce inhibitory signals that maintain the balance between T cell activation and tolerance [26]. Wang noted that reduced expression of PD-1 and Tim-3 on decidual CD4^+^ T cells in mice might be linked with miscarriage [28]. The relations between T cells, APC, and trophoblast cells’ immune checkpoint expression are illustrated in Figure 1 and Figure 2.

During pregnancy, a special type of immunological process occurs [7]. To achieve a successful pregnancy, it is necessary to keep an immunological balance between mother and fetus. A mother’s immunity allows normal trophoblast invasion, which is a semi-allograft from an immunological point of view [7,29]. Dysregulation of maternal–fetal immunity is significantly linked to pregnancy loss [30,31]. 

Miscarriage is a fatal complication of pregnancy, and the problem is increasing worldwide. If there are more than two miscarriages, it is defined as recurrent spontaneous abortion (RSA) [31]. Despite well-described causes of RSA such as chromosomal abnormalities, uterine anatomical malformations, endocrine dysfunctions, thrombophilic factors, and immune disorders, in approximately 50% of RSA cases, the reasons remain unknown [32]. Currently, increasing numbers of studies correlate recurrent miscarriages with alterations in the maternal immunological system [32]. Still, the immunological crosstalk between the maternal immune system and fetal cells is poorly understood, and studies in these area remain necessary [29]. In this regard, finding differences in expression of ICP molecules and better understanding the role of co-inhibitory molecules and their immunoregulatory function may be utilized in the diagnosis and treatment of pregnancy loss. In line with this thought, our study aimed to determine the expression of two major immune checkpoint receptors, TIM-3 and PD-1, on T lymphocyte subpopulations and NK and NKT cells of non-pregnant women with a history of RSA in comparison with non-pregnant healthy, fertile women. Studies were performed on freshly isolated peripheral blood to prevent the influence of storage (freezing and thawing) on the structure of the molecule. 

## 2. Materials and Methods

### 2.1. Ethical Considerations

The study was approved by the Bioethics Committee of the Medical University of Warsaw. Number of Bioethics Committee approval: KB/13/2020. All measurements, interventions, and blood collections were performed after obtaining informed consent. All the procedures were followed in accordance with the Helsinki Declaration of 1975, as revised in 2013.

### 2.2. Study Group

Twenty-four patients with RSA were included in the study group. The recurrent pregnancy loss was defined as two or more consecutive spontaneous miscarriages before the 20th week of gestation. All patients included in the study were at least 6 months after the last miscarriage, so the immunological status of patients was normalized before the research. Moreover, the study group had peripheral blood chromosome assessments that revealed normal karyotypes. Patients with anatomic, genetic, microbiological, immunological, and hormonal causes of abortions were excluded from the research. 

### 2.3. Control Group

The control group consisted of 12 fertile, non-pregnant women without disorders in their obstetric–gynecological and internal medicine histories. All women in the control group had had at least one childbirth without complication; all subjects declared a normal course of pregnancy and delivery. None of the women in the control group had had a miscarriage. In addition, none of the control subjects had been treated for any internal disorders.

### 2.4. Cell Preparation

Blood was vested in heparin tubes. PBMCs were isolated by gradient centrifugation, 800× *g*, 12 min on Histopaque-1077 (MERCK, Darmstadt, Germany). The cells from the interphase were harvested and washed in phosphate-buffered saline (PBS) (Aqua-Med, Łódź, Poland) 600× *g*, 10 min. This step was repeated twice. Then cells were resuspended in RPMI enriched with 10% heat-inactivated fetal calf serum (MERCK, Darmstadt, Germany), 2 mM glutamine (MERCK, Darmstadt, Germany), antibiotic-antimycotic solution 100 I.U., penicillin, 100 µg/mL Streptomycin, and 0.25 µg/mL Amphotericin (Corning, New York, BD, USA).

### 2.5. Flow Cytometry Staining

Cells were counted in the light microscope; 1 × 10^6^ cells were used for staining. Cells were washed by centrifugation in 1 mL PBS without Ca^2+^ and Mg^2+^ with 0.01% sodium azide (Aqua-Med, Łódź, Poland). Cells were resuspended in 100 μL PBS without Ca^2+^ and Mg^2+^ with 0.01% sodium azide and stained with antibodies against specific surface antigens: CD4-APC-Cy7 (SK3 clone, Becton Dickinson, Franklin Lakes, NJ, USA), CD3-PerCP (SK7 clone, Becton Dickinson, USA), CD8-APC (SK1 clone, Becton Dickinson, USA), CD56-PE-Cy7 (B159 clone, Becton Dickinson, Franklin Lakes, NJ, USA), CD279-BV480 (EH12.1 clone, Becton Dickinson, Franklin Lakes, NJ, USA), and CD366-PE (7D3 clone, Becton Dickinson, Franklin Lakes, NJ, USA). Samples were incubated in the dark for 15 min, room temperature. Next, cells were rinsed in 1 mL of PBS without Ca2^+^ and Mg2^+^ with 0.01% sodium azide, 2000 rpm, 5 min, then resuspended in 200 μL PBS without Ca^2+^ and Mg^2+^ with 0.01% sodium azide. The gating strategy was established based on fluorescence minus one (FMO) experiments (please refer to Supplementary Materials, Figures S1 and S2). A Becton Dickinson FACSCanto II cytometer (BD FACS Canto II, Becton Dickinson, Franklin Lakes, NJ, USA) was used to collect data, then analysis was performed with BD FACS Diva 6.1.3. software. Relative frequencies of TIM-3 and PD-1 expression were assessed on T helper lymphocytes (CD3^+^CD4^+^), T cytotoxic lymphocytes (CD3^+^CD8^+^), NK cells (CD3^-^CD56^+^), and NKT cells (CD3^+^ CD56^+^).

### 2.6. Statistical Analyses

All statistical analyses were performed with Graph Pad Prism 8.4.1, and the results were shown as mean plus/minus standard deviation (SD). Gaussian distribution was determined with the Shapiro–Wilk test. The analyses between sets of data with Gaussian distribution were performed considering the F-test. For groups with the same SD, the unpaired t-test was used. For groups with different SDs, an unpaired t-test with Welch’s correction was used. The analyses between groups without Gaussian distribution were performed with the Mann–Whitney test. P values below 0.05 (*p* < 0.05) were considered statistically significant and marked on the graphs as *.

## 3. Results

**Figure 3:**
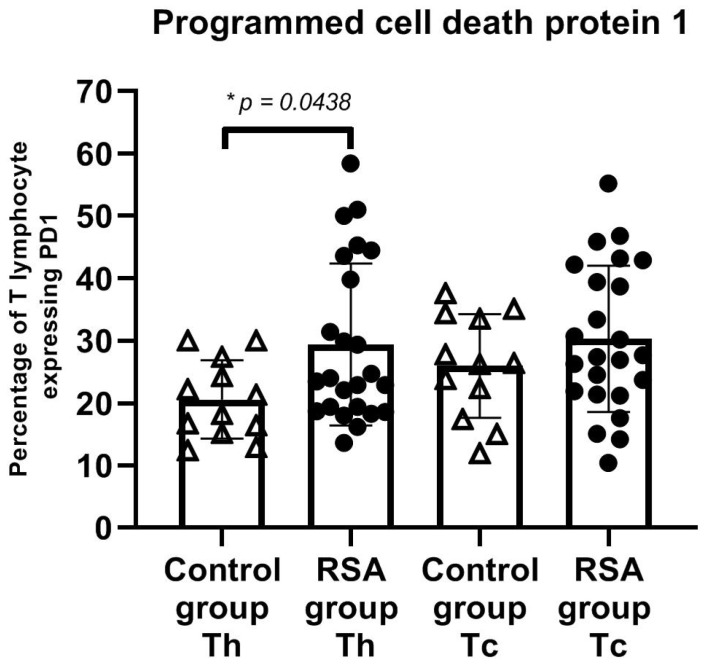
Expression of PD-1 on T cell subsets. A higher percentage of PD-1 expression on T helper lymphocytes was shown in the RSA group. The error bars represent the standard deviation (SD). Abbreviations: RSA group—women with recurrent spontaneous abortion, control group—healthy fertile women, Th—helper T lymphocyte (CD4+), Tc—cytotoxic T lymphocyte (CD8+), * *p* < 0.05 (RSA group n = 24, control group n = 12). Please refer to the Supplementary Information for gating strategy (Figures S1 and S2).

**Figure 4:**
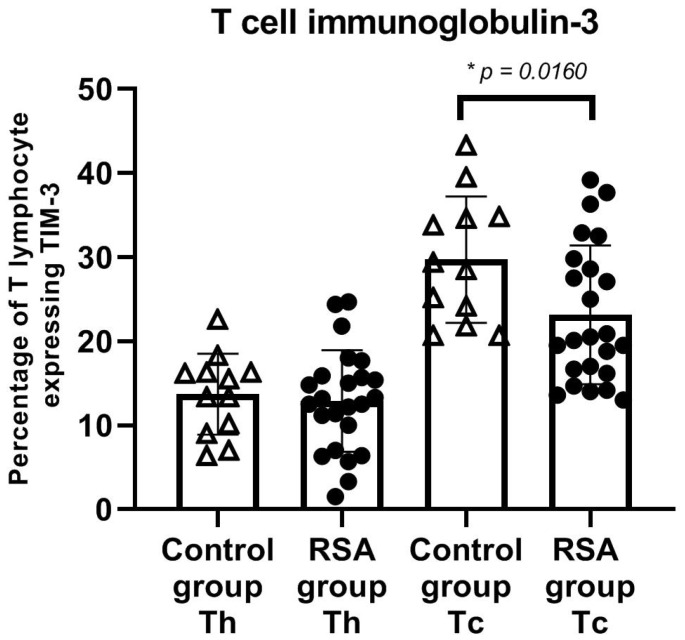
Percentage expression of TIM-3 on cells subsets. A higher percentage of TIM-3 expression on cytotoxic T lymphocytes was shown in RSA than in the control group. The error bars represent the standard deviation (SD). Abbreviations: RSA group—women with recurrent spontaneous abortion, control group—healthy fertile women, Th—helper T lymphocyte (CD4+), Tc—cytotoxic T lymphocyte (CD8+), * *p* < 0.05 (RSA group n = 24, control group n = 12). Please refer to the Supplementary Information for gating strategy (Figures S1 and S2).

We found significantly higher expression of PD-1 on T helper (CD4+) cells in women with RSA (*p* < 0.05). Elevated expression of PD-1 expanded on all studied lymphocyte populations in the RSA group (Figure 3). Significant differences in TIM-3 expression were found on T cytotoxic phenotype (CD8+) of lymphocytes (Figure 4), where the expression was emphasized in the control group. The expression of studied ICPs was similar between groups considering NK and NKT cells.

## 4. Discussion

During the physiological development of pregnancy, from a very early stage, significant changes in the maternal immune system occur to tolerate the presence of the fetus and provide an optimal environment. In our study, we demonstrate that PD-1 expression on CD4^+^ lymphocytes in women with RSA was elevated in comparison to the healthy women, which may suggest that expression of cytokines or chemokine involved in pregnancy progression is excessively suppressed. 

Meggyes et al. found that PD-1 expression on peripheral Tc and Th lymphocytes was decreased in the first trimester of pregnant women in comparison to non-pregnant women [24]. The Meggyes et al. findings suggest that, during the first trimester of pregnancy, the immune system is inhibited, and this change is necessary to warrant proper development of the pregnancy [24]. Further functional studies of immune cells are necessary to prove this suggestion. Moreover, Wang et al. observed a tremendous decrease in PD-1 and TIM-3 expression on Th cells of RSA women at the time of miscarriage; however, researchers did not notice changes in expression of a single population of TIM-3 or PD-1 positive lymphocytes [28]. Wang et al., in their recent study considering women with RSA, show that expression of PD-1 was significantly decreased on Th1 and Th17 cells, but not on Treg cells [33]. Hofmeyer et al. and Blackburn et al., similarly to our studies, found that overexpression of PD-1 can cause T cell dysfunction and exhaustion accompanied by impaired IFN-γ secretion [34,35]. Moreover, PD-1 activation may impair effector T cell proliferation and function while promoting regulatory T cell differentiation and its suppression function [7,36]. Overexpression of dysfunctional Treg cells was reported in women with RSA and preeclampsia [37]. In addition, in our previous study performed on a similar group of patients, we noticed significantly impaired Th1 cytokine production in RSA patients compared to fertile women, which may suggest immune system exhaustion [38]. Thus, we can speculate that blockade of PD-1 molecule could restore immune-cell function and help to achieve positive pregnancy outcomes in RSA patients with an exhausted immune system. 

The second ICP molecule studied in our research was TIM-3. Enninga et al. found that the level of soluble Gal-9 increases throughout pregnancy [39]. Decreased expression of TIM-3 probably leads to impaired interaction with Gal-9 in women with RSA, which causes Th1/Th2 and Tc1/Tc2 dysregulation. On the other hand, decreased expression of immune checkpoints might be correlated with elevated cytotoxicity of Tc cells [15,40]. We observed a significant decrease in TIM-3 on Tc cells in RSA patients. Wang et al., which demonstrated that Tim-3 blockade, performed in a mouse-pregnancy model (female CBA/J × male BALB/c), caused higher susceptibility to fetal loss, and was associated with Th1/Th2 and Tc1/Tc2 imbalances in the cytokine production from CD4^+^ and CD8^+^ T cells [28,41]. Chabtini et al. demonstrated that Tim-3 blockade in the female CBA × male B6 mouse model caused ex vivo upregulation of proinflammatory IFN-γ and TNF-α cytokine production [19]. Considering previously mentioned studies, decreased expression of TIM-3 on Tc lymphocytes in our study group may lead to fetal loss. Moreover, Wang et al. found that the number of TIM-3^+^ cytotoxic T lymphocytes is decreased in miscarriage patients compared to pregnant women [42], which corresponds with our results. At the maternal–fetal interface, CD8 T cells should not exist as exhausted cells but show high proliferative activity and an anti-inflammatory cytokine profile [15]. The blockade of TIM-3 in vitro results in impaired proliferation of CD8+ T cells and shifts their cytokine profile to an increase in IFN-γ production and cytotoxic capacity. These changes can cause trophoblast cell death [42]. Zhuang et al. assessed TIM-3 mRNA expression in PBMCs and Th1 cells obtained from RSA patients and normal pregnant females. They found that TIM-3 expression in PBMCs was significantly higher in the RSA group in comparison to normal pregnant women [43]. Flow cytometry results obtained in this experiment reveal that the ratio of the CD4+TIM3+/CD4+ cells in PBMCs was significantly higher in the RSA group than in the control group [43]. We did not notice such correlation and changes in TIM-3 expression on CD4+ cells in our studied groups. 

The most abundant population of immune cells in MFI are NK cells. Sun et al. reported a strong correlation with the protector function of decidual NK cells and TIM-3 expression during the first trimester of pregnancy; furthermore, TIM-3-positive NK cells were mainly found in decidua, not in peripheral blood [44]. Decidual TIM-3^+^ NK cells display a Th2 profile with low cytotoxicity [45,46]. Unfortunately, there are no data describing differences in expression of TIM3 or PD-1 on pbNK cells between non-pregnant RSA and fertile women. Nevertheless, our research suggests a lack of alterations in studied molecules in non-pregnant fertile and RSA women. 

Our research sheds new light on the role of ICPs in reproductive immunology. Further exploration of this area may also allow for a more detailed explanation of the previously known disorders (shift of cytokine production toward Th1 profile, increased cytotoxicity, and proliferation of immune cells) during pregnancy. 

The topic of this work requires further investigation, since this study is limited by the small group size and the inclusion of only two membrane ICPs determined only on main T cell subsets. A study of ICP ligands and soluble counterparts of ICPs and their ligands will be valuable. The TIM-3 and PD-1 represent two of many immune checkpoints; thus, additional studies on a larger variety of co-inhibitory molecules (LAG-3, VISTA, TIGIT, CTLA-4, BTLA) might elucidate the role and function of ICP molecules in maternal–fetal immune interactions. To define whether the expression of ICPs is associated with a miscarriage, it would be useful to examine women at the time of the miscarriage to reflect their immune status during the pathological process, in comparison to women who develop normal pregnancies. The wider analysis of ICPs may also yield new biomarkers for the diagnosis and prevention of RSA.

## 5. Conclusions

Our observations indicate that specific shifts in expression of TIM-3 and PD-1 might either be related to the pathogenesis of recurrent spontaneous abortions or be a repercussion of them. Nevertheless, differences that we observed in TIM-3 expression led to the conclusion that specific RSA women’s lymphocytes are shifted toward Tc1 and impaired Th response in comparison to fertile women. Further studies of the expression and function of ICPs in RSA women might shed new light on the reasons for pregnancy loss, as well as provide new trends in therapy and diagnosis.

## Data Availability

The PD1Tim3.xlsx data used to support the findings of this study are available from the corresponding author upon request.

## Supplementary Materials

The following are available online at https://www.mdpi.com/article/10.3390/jcm10184182/s1, Figure S1: Graph 1, Figure S2: Graph 2.

## References

[1] Marhelava K., Pilch Z., Bajor M., Graczyk-Jarzynka A., Zagozdzon R. (2019). Targeting Negative and Positive Immune Checkpoints with Monoclonal Antibodies in Therapy of Cancer. Cancers.

[2] Joller N., Kuchroo V.K. (2017). Tim-3, Lag-3, and TIGIT. Curr. Top. Microbiol. Immunol..

[3] Schildberg F.A., Klein S.R., Freeman G.J., Sharpe A.H. (2016). Coinhibitory Pathways in the B7-CD28 Ligand-Receptor Family. Immunity.

[4] Wing K., Onishi Y., Prieto-Martin P., Yamaguchi T., Miyara M., Fehervari Z., Nomura T., Sakaguchi S. (2008). CTLA-4 control over Foxp3+ regulatory T cell function. Science.

[5] Gleason M.K., Lenvik T.R., McCullar V., Felices M., O’Brien M.S., Cooley S.A., Verneris M.R., Cichocki F., Holman C.J., Panoskaltsis-Mortari A. (2012). Tim-3 is an inducible human natural killer cell receptor that enhances interferon gamma production in response to galectin-9. Blood.

[6] Zhang Y.H., Sun H.X. (2020). Immune checkpoint molecules in pregnancy: Focus on regulatory T cells. Eur. J. Immunol..

[7] Xu Y.Y., Wang S.C., Li D.J., Du M.R. (2017). Co-Signaling Molecules in Maternal-Fetal Immunity. Trends Mol. Med..

[8] Huang Y.H., Zhu C., Kondo Y., Anderson A.C., Gandhi A., Russell A., Dougan S.K., Petersen B.S., Melum E., Pertel T. (2015). CEACAM1 regulates TIM-3-mediated tolerance and exhaustion. Nature.

[9] Zhu C., Anderson A.C., Schubart A., Xiong H., Imitola J., Khoury S.J., Zheng X.X., Strom T.B., Kuchroo V.K. (2005). The Tim-3 ligand galectin-9 negatively regulates T helper type 1 immunity. Nat. Immunol..

[10] Kang C.W., Dutta A., Chang L.Y., Mahalingam J., Lin Y.C., Chiang J.M., Hsu C.Y., Huang C.T., Su W.T., Chu Y.Y. (2015). Apoptosis of tumor infiltrating effector TIM-3+CD8+ T cells in colon cancer. Sci. Rep..

[11] Santiago C., Ballesteros A., Martinez-Munoz L., Mellado M., Kaplan G.G., Freeman G.J., Casasnovas J.M. (2007). Structures of T cell immunoglobulin mucin protein 4 show a metal-Ion-dependent ligand binding site where phosphatidylserine binds. Immunity.

[12] Cao E., Zang X., Ramagopal U.A., Mukhopadhaya A., Fedorov A., Fedorov E., Zencheck W.D., Lary J.W., Cole J.L., Deng H. (2007). T Cell Immunoglobulin Mucin-3 Crystal Structure Reveals a Galectin-9-Independent Ligand-Binding Surface. Immunity.

[13] Chiba S., Baghdadi M., Akiba H., Yoshiyama H., Kinoshita I., Dosaka-Akita H., Fujioka Y., Ohba Y., Gorman J.V., Colgan J.D. (2012). Tumor-infiltrating DCs suppress nucleic acid-mediated innate immune responses through interactions between the receptor TIM-3 and the alarmin HMGB1. Nat. Immunol..

[14] Miko E., Meggyes M., Doba K., Barakonyi A., Szereday L. (2019). Immune Checkpoint Molecules in Reproductive Immunology. Front. Immunol..

[15] Meggyes M., Miko E., Polgar B., Bogar B., Farkas B., Illes Z., Szereday L. (2014). Peripheral blood TIM-3 positive NK and CD8+ T cells throughout pregnancy: TIM-3/galectin-9 interaction and its possible role during pregnancy. PLoS ONE.

[16] Sanchez-Fueyo A., Tian J., Picarella D., Domenig C., Zheng X.X., Sabatos C.A., Manlongat N., Bender O., Kamradt T., Kuchroo V.K. (2003). Tim-3 inhibits T helper type 1-mediated auto- and alloimmune responses and promotes immunological tolerance. Nat. Immunol..

[17] Hu X.H., Tang M.X., Mor G., Liao A.H. (2016). Tim-3: Expression on immune cells and roles at the maternal-fetal interface. J. Reprod. Immunol..

[18] Shimizu Y., Kabir-Salmani M., Azadbakht M., Sugihara K., Sakai K., Iwashita M. (2008). Expression and localization of galectin-9 in the human uterodome. Endocr. J..

[19] Chabtini L., Mfarrej B., Mounayar M., Zhu B., Batal I., Dakle P.J., Smith B.D., Boenisch O., Najafian N., Akiba H. (2013). TIM-3 regulates innate immune cells to induce fetomaternal tolerance. J. Immunol..

[20] Miko E., Meggyes M., Bogar B., Schmitz N., Barakonyi A., Varnagy A., Farkas B., Tamas P., Bodis J., Szekeres-Bartho J. (2013). Involvement of Galectin-9/TIM-3 pathway in the systemic inflammatory response in early-onset preeclampsia. PLoS ONE.

[21] Li J., Li F.F., Zuo W., Zhou Y., Hao H.Y., Dang J., Jiang M., He M.Z., Deng D.R. (2014). Up-regulated expression of Tim-3/Gal-9 at maternal-fetal interface in pregnant woman with recurrent spontaneous abortion. J. Huazhong Univ. Sci. Technolog. Med. Sci..

[22] Hao H., He M., Li J., Zhou Y., Dang J., Li F., Yang M., Deng D. (2015). Upregulation of the Tim-3/Gal-9 pathway and correlation with the development of preeclampsia. Eur. J. Obstet. Gynecol. Reprod. Biol..

[23] Wu M., Zhu Y., Zhao J., Ai H., Gong Q., Zhang J., Zhao J., Wang Q., La X., Ding J. (2015). Soluble costimulatory molecule sTim3 regulates the differentiation of Th1 and Th2 in patients with unexplained recurrent spontaneous abortion. Int. J. Clin. Exp. Med..

[24] Meggyes M., Miko E., Szigeti B., Farkas N., Szereday L. (2019). The importance of the PD-1/PD-L1 pathway at the maternal-fetal interface. BMC Pregnancy Childbirth.

[25] Freeman G.J., Long A.J., Iwai Y., Bourque K., Chernova T., Nishimura H., Fitz L.J., Malenkovich N., Okazaki T., Byrne M.C. (2000). Engagement of the PD-1 immunoinhibitory receptor by a novel B7 family member leads to negative regulation of lymphocyte activation. J. Exp. Med..

[26] Keir M.E., Butte M.J., Freeman G.J., Sharpe A.H. (2008). PD-1 and its ligands in tolerance and immunity. Annu. Rev. Immunol..

[27] Sledzinska A., Menger L., Bergerhoff K., Peggs K.S., Quezada S.A. (2015). Negative immune checkpoints on T lymphocytes and their relevance to cancer immunotherapy. Mol. Oncol..

[28] Wang S., Zhu X., Xu Y., Zhang D., Li Y., Tao Y., Piao H., Li D., Du M. (2016). Programmed cell death-1 (PD-1) and T-cell immunoglobulin mucin-3 (Tim-3) regulate CD4+ T cells to induce Type 2 helper T cell (Th2) bias at the maternal-fetal interface. Hum. Reprod..

[29] Trowsdale J., Betz A.G. (2006). Mother’s little helpers: Mechanisms of maternal-fetal tolerance. Nat. Immunol..

[30] Rai R., Regan L. (2006). Recurrent miscarriage. Lancet.

[31] Williams Z. (2012). Inducing tolerance to pregnancy. N. Engl. J. Med..

[32] Mekinian A., Cohen J., Alijotas-Reig J., Carbillon L., Nicaise-Roland P., Kayem G., Darai E., Fain O., Bornes M. (2016). Unexplained Recurrent Miscarriage and Recurrent Implantation Failure: Is There a Place for Immunomodulation?. Am. J. Reprod. Immunol..

[33] Wang W.J., Salazar Garcia M.D., Deutsch G., Sung N., Yang X., He Q., Jubiz G., Bilal M., Dambaeva S., Gilman-Sachs A. (2020). PD-1 and PD-L1 expression on T-cell subsets in women with unexplained recurrent pregnancy losses. Am. J. Reprod. Immunol..

[34] Blackburn S.D., Shin H., Haining W.N., Zou T., Workman C.J., Polley A., Betts M.R., Freeman G.J., Vignali D.A., Wherry E.J. (2009). Coregulation of CD8+ T cell exhaustion by multiple inhibitory receptors during chronic viral infection. Nat. Immunol..

[35] Hofmeyer K.A., Jeon H., Zang X. (2011). The PD-1/PD-L1 (B7-H1) pathway in chronic infection-induced cytotoxic T lymphocyte exhaustion. J. Biomed. Biotechnol..

[36] Gupta S., Thornley T.B., Gao W., Larocca R., Turka L.A., Kuchroo V.K., Strom T.B. (2012). Allograft rejection is restrained by short-lived TIM-3+PD-1+Foxp3+ Tregs. J. Clin. Investig..

[37] Lamprianidou E., Daniilidis M., Kordella C., Zoulia E., Nakou E., Gerofotis A., Vasilaki A., Pantos G., Kotsianidis I. (2020). The STAT signaling profile at the single cell level reveals novel insights in the association of FOXP3+ T regulatory cells with recurrent spontaneous abortions before and after lymphocyte immunotherapy. Clin. Immunol..

[38] Kniotek M., Zych M., Roszczyk A., Szafarowska M., Jerzak M.M. (2021). Decreased Production of TNF-α and IL-6 Inflammatory Cytokines in Non-Pregnant Idiopathic RPL Women Immunomodulatory Effect of Sildenafil Citrate on the Cellular Response of Idiopathic RPL Women. J. Clin. Med..

[39] Enninga E.A.L., Harrington S.M., Creedon D.J., Ruano R., Markovic S.N., Dong H., Dronca R.S. (2018). Immune checkpoint molecules soluble program death ligand 1 and galectin-9 are increased in pregnancy. Am. J. Reprod. Immunol..

[40] Meggyes M., Nagy D.U., Szereday L. (2020). Investigation of the PD-1 and PD-L1 Immune Checkpoint Molecules Throughout Healthy Human Pregnancy and in Nonpregnant Women. J. Clin. Med..

[41] Wang S., Cao C., Piao H., Li Y., Tao Y., Zhang X., Zhang D., Sun C., Zhu R., Wang Y. (2015). Tim-3 protects decidual stromal cells from toll-like receptor-mediated apoptosis and inflammatory reactions and promotes Th2 bias at the maternal-fetal interface. Sci. Rep..

[42] Wang S.C., Li Y.H., Piao H.L., Hong X.W., Zhang D., Xu Y.Y., Tao Y., Wang Y., Yuan M.M., Li D.J. (2015). PD-1 and Tim-3 pathways are associated with regulatory CD8+ T-cell function in decidua and maintenance of normal pregnancy. Cell Death Dis..

[43] Zhuang X., Xia X., Liu L., Zhang Y., Zhang X., Wang C. (2018). Expression of Tim-3 in peripheral blood mononuclear cells and placental tissue in unexplained recurrent spontaneous abortion. Medicine.

[44] Sun J., Yang M., Ban Y., Gao W., Song B., Wang Y., Zhang Y., Shao Q., Kong B., Qu X. (2016). Tim-3 Is Upregulated in NK Cells during Early Pregnancy and Inhibits NK Cytotoxicity toward Trophoblast in Galectin-9 Dependent Pathway. PLoS ONE.

[45] Li Y., Zhang J., Zhang D., Hong X., Tao Y., Wang S., Xu Y., Piao H., Yin W., Yu M. (2017). Tim-3 signaling in peripheral NK cells promotes maternal-fetal immune tolerance and alleviates pregnancy loss. Sci. Signal..

[46] Li Y.H., Zhou W.H., Tao Y., Wang S.C., Jiang Y.L., Zhang D., Piao H.L., Fu Q., Li D.J., Du M.R. (2016). The Galectin-9/Tim-3 pathway is involved in the regulation of NK cell function at the maternal-fetal interface in early pregnancy. Cell. Mol. Immunol..

