# Supplementary material for: Differences in Immune Checkpoints Expression (TIM-3 and PD-1) on T Cells in Women with Recurrent Miscarriages—Preliminary Studies
Source: J Clin Med. 2021 Sep 16;10(18):4182. doi: 10.3390/jcm10184182 (PMC8468868; doi:10.3390/jcm10184182)
Supplement: Supplementary file 1 [file jcm-10-04182-s001.zip › jcm-1328045-supplementary.pdf]

Journal of
Clinical Medicine

MDPI

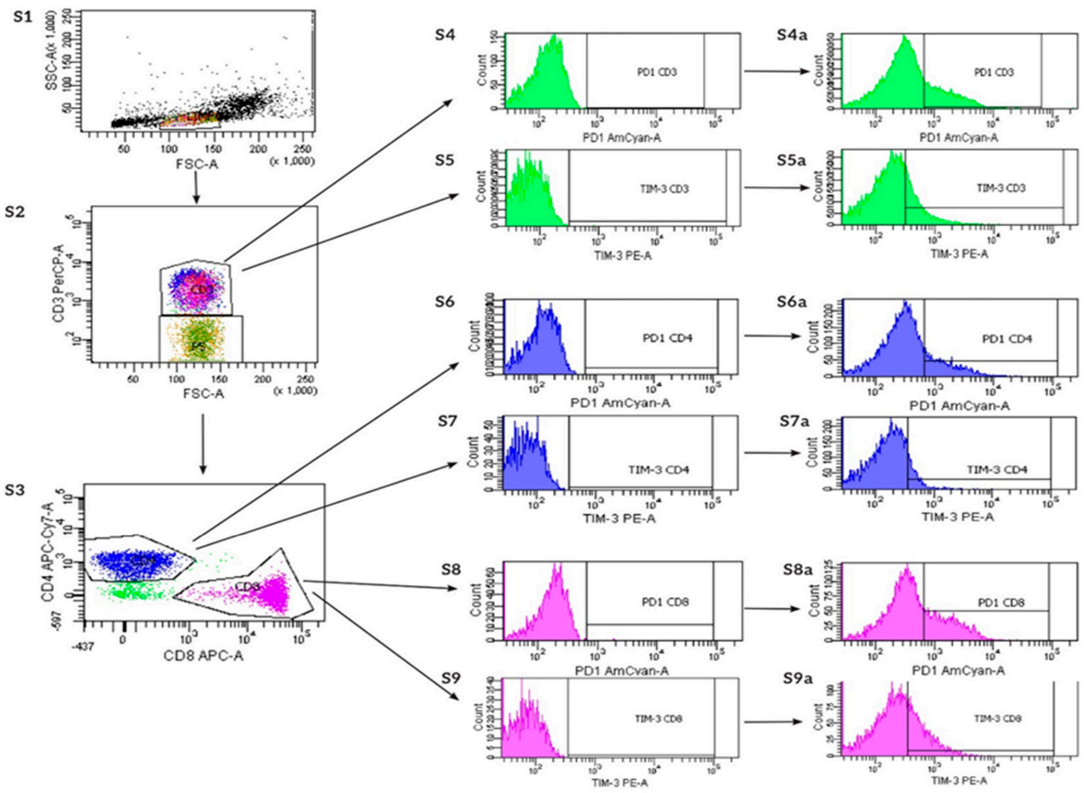

Figure S1. Graph 1-Gating strategy: lymphocytes were identified on FSC/SSC dot plot S1) as cells within 50-100 x 10³ on FSC and below 50 x 10³ on SSC. S2) CD3 positive cells (T lymphocytes were identified as events with PreCP fluorescence above 10³ on logarithmic scale) S3) CD4 positive cells (T helper lymphocytes were identified as events with APC-Cy7 fluorescence above 10² on logarithmic scale) and CD8 positive cells (T cytotoxic lymphocytes were identified as events with APC fluorescence above 10³ on logarithmic scale) S4-S9)Cut-off values for PD1 (AmCyan channel) and TIM3 (PE channel) considering each studied population (total T cell, T helper and T cytotoxic cells) were established basing on fluorescence minus one (FMO) experiments, performed each time test was run. S4a-S9a) PD1 and Tim-3 positive populations were identified with fluorescence higher than fluorescence minus one, therefore positive.

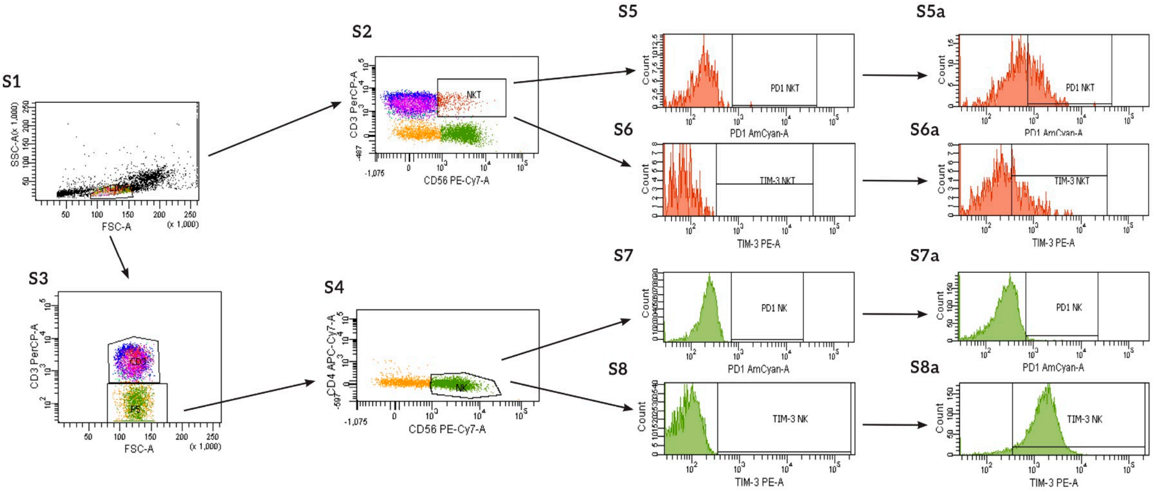

Figure S2. Graph 2-Gating strategy: lymphocytes were identified on FSC/SSC dot plot S1) as cells within 50-100 x 10³ on FSC and below 50 x 10³ on SSC, S2) NKT cells were identified as CD56 positive(fluorescence above 10³ Pe-Cy-7 channel, logarithmic scale) and CD 3 positive (fluorescence above 10³ PerCP channel, logarithmic scale), S3) CD3 negative cells

(non- T lymphocytes were identified as events with PreCP fluorescence below 10^3 on logarithmic scale), S4) NK cells were identified as CD56 positive(fluorescence above 10^3 Pe-Cy-7 channel, logarithmic scale) and negative for CD3(S3), S5-S8)Cut-off values for PD1 (AmCyan channel) and TIM3 (PE channel) considering each studied population (NKT and NK cells) were established basing on fluorescence minus one (FMO) experiments, performed each time test was run. S5a-S8a) PD1 and Tim-3 positive populations were identified with fluorescence higher than fluorescence minus one, therefore positive.
